# Survey of the Impact of COVID-19 on Oncologists’ Decision Making in Cancer

**DOI:** 10.1200/GO.20.00300

**Published:** 2020-08-05

**Authors:** Yüksel Ürün, Syed A. Hussain, Ziad Bakouny, Daniel Castellano, Saadettin Kılıçkap, Gilberto Morgan, Rana R. Mckay, Kevin Pels, Andrew Schmidt, Deborah B. Doroshow, Fábio Schütz, Laurence Albiges, Gilberto Lopes, James W. F. Catto, Solange Peters, Toni K. Choueiri

**Affiliations:** ^1^ Department of Medical Oncology, Ankara University School of Medicine, Ankara, Turkey; ^2^ Ankara University Cancer Research Institute, Ankara, Turkey; ^3^ Academic Unit of Oncology, University of Sheffield, Sheffield, United Kingdom; ^4^ Department of Medical Oncology, Lank Center for Genitourinary Oncology, Dana-Farber Cancer Institute, Harvard Medical School, Boston, MA; ^5^ Medical Oncology Department, Hospital Universitario 12 de Octubre, Madrid, Spain; ^6^ Hacettepe University Cancer Institute, Ankara, Turkey; ^7^ Department of Medical and Radiation Oncology, Skåne University Hospital, Lund, Sweden; ^8^ Moores Cancer Center, University of California San Diego, La Jolla, CA; ^9^ Tisch Cancer Institute, Icahn School of Medicine at Mount Sinai, New York, NY; ^10^ Clinical Oncology Department, BP - A Beneficência Portuguesa de São Paulo, São Paulo, Brazil; ^11^ Department of Medical Oncology, Gustave Roussy Cancer Campus, Villejuif, France; ^12^ Division of Hematology and Medical Oncology, Department of Medicine, Miller School of Medicine, University of Miami, Sylvester Comprehensive Cancer Center, Miami, FL; ^13^ Academic Urology Unit, University of Sheffield, Sheffield, United Kingdom; ^14^ Oncology Department, Centre Hospitalier Universitaire Vaudois, Lausanne University, Lausanne, Switzerland

## Abstract

**PURPOSE:**

To understand readiness measures taken by oncologists to protect patients and health care workers from the novel coronavirus (COVID-19) and how their clinical decision making was influenced by the pandemic.

**METHODS:**

An online survey was conducted between March 24 and April 29, 2020.

**RESULTS:**

A total of 343 oncologists from 28 countries participated. The median age was 43 years (range, 29-68 years), and the majority were male (62%). At the time of the survey, nearly all participants self-reported an outbreak in their country (99.7%). Personal protective equipment was available to all participants, of which surgical mask was the most common (n = 308; 90%). Telemedicine, in the form of phone or video encounters, was common and implemented by 80% (n = 273). Testing patients with cancer for COVID-19 via reverse transcriptase polymerase chain reaction before systemic treatment was not routinely implemented: 58% reported no routine testing, 39% performed testing in selected patients, and 3% performed systematic testing in all patients. The most significant factors influencing an oncologist’s decision making regarding choice of systemic therapy included patient age and comorbidities (81% and 92%, respectively). Although hormonal treatments and tyrosine kinase inhibitors were considered to be relatively safe, cytotoxic chemotherapy and immune therapies were perceived as being less safe or unsafe by participants. The vast majority of participants stated that during the pandemic they would use less chemotherapy, immune checkpoint inhibitors, and steroids. Although treatment in neoadjuvant, adjuvant, and first-line metastatic disease was less affected, most of the participants stated that they would be more hesitant to recommend second- or third-line therapies in metastatic disease.

**CONCLUSION:**

Decision making by oncologists has been significantly influenced by the ongoing COVID-19 pandemic.

## INTRODUCTION

The severe acute respiratory syndrome coronavirus 2 (SARS-CoV-2) viral pandemic has affected nearly all sectors of health care globally.^1,2^ As of June 17, 2020, > 8.2 million people have been diagnosed with the novel coronavirus (COVID-19), and 430,000 have died as a result of the disease worldwide.^3^

**CONTEXT**

**Key Objective**
Does COVID-19 influence the decision-making process of oncologists?
**Knowledge Generated**
In this international survey including 343 oncologists from 28 countries, the most commonly used personal protective equipment was the surgical mask. Telemedicine is being increasingly used. The most significant factors influencing an oncologist’s decision making regarding the determination of treatment were patient age and comorbidities. Hormonal treatments and tyrosine kinase inhibitors were considered to be relatively safe, but cytotoxic and immune therapies were perceived as being less safe or unsafe by respondents. Likewise, neoadjuvant, adjuvant, and first-line metastatic disease was less affected, but most of the participants stated that they would be more reluctant to recommend second- or third-line therapies in the metastatic setting
**Relevance**
During the pandemic, the decision-making process of oncologists is significantly affected. International collaboration and prospective studies are critical in providing a stronger evidentiary basis for making these decisions.

COVID-19 has had a large and negative impact on cancer treatment and research.^5,6^ There is significant concern that the pandemic could lead to adverse outcomes related to other preexisting conditions, including cancer. This concern is driven by the potential for delayed presentation, diagnosis, and/or treatment that could emanate from patient avoidance of hospital visits, doctors’ assumptions about the risk/benefit ratio of every intervention, as well as health care resource reallocation to patients with COVID-19.^6-12^ In addition, COVID-19 has already had an impact on cancer research.

Patients with cancer are considered to be at increased risk from COVID-19–related complications because of treatment-related immunosuppression, increased comorbidities, and the underlying malignancy itself.^9,13-19^ In addition, they may be more likely to contract COVID-19 secondary to frequent contact with the health system and a high-risk environment for COVID transmission.^20,21^ Organizations such as the European Society for Medical Oncology (ESMO), ASCO, The National Comprehensive Cancer Network, and the American Association for Cancer Research (AACR) have published guidelines regarding the precautions and treatment modifications during the pandemic.^22-26^ We must carefully weigh the uncertainty from the additional risk of infection versus benefit from treatment. Although we still ignore the specific vulnerability resulting from various oncological scenarios as well as the variety of anticancer strategies, we do not have adequate knowledge on the long-term impact of current changes in oncologic practice.^26-28^

In this international, web-based survey, oncologists were asked about pandemic-related changes in their clinical practices and personal measures taken to protect their own physical well-being in response to the COVID-19 pandemic.

## METHODS

### Study Design

We conducted a global survey of medical oncologists. Respondents were contacted through differing distribution channels, including direct e-mail and social media networks such as Twitter and oncology-specific groups on Facebook. The survey was conducted between March 24 and April 29, 2020. Data collected included demographics, country, practice setting, and years of experience. In addition, the survey tool included questions regarding attitudes of medical oncologists around patient risk factors (age, performance status, comorbidities), administration of types of antineoplastic therapy (cytotoxic therapy, targeted therapy, immunotherapy), and use of therapy in differing settings (neoadjuvant, adjuvant *v* metastatic). The question “Do you perform COVID-19 RT-PCR test before the treatment” was added to the questionnaire on April 11, 2020.

All the data in this survey are collected anonymously, with no personal information (apart from their name and publicly available contact details). The full survey can be accessed at: https://docs.google.com/forms/d/e/1FAIpQLSerbTv8Bi6mlw6Cfuh9cJTLJWgPP9jP4jBp4s4qc5hfz9F9SA/viewform.

### Statistical Analysis

The frequencies of all categorical data were calculated. Bar plots and stacked bar plots were used to visualize the data. All statistical analyses were carried out using SPSS version 21.0 (SSPS, Chicago, IL).

## RESULTS

### Participant Demographics

A total of 343 oncologists from 28 countries participated in the survey, and 95% of responses were received between April 1 and April 29, 2020. The median age of the participants was 43 years (range, 29-68 years), and the majority were male (62%). At the time of the survey, almost all participants stated that there was an outbreak in their country (99.7%). Most of the participants practiced at a university or academic center (71%) and have > 10 years of experience in practice (65%; Tables 1 and 2).

**TABLE 1:**
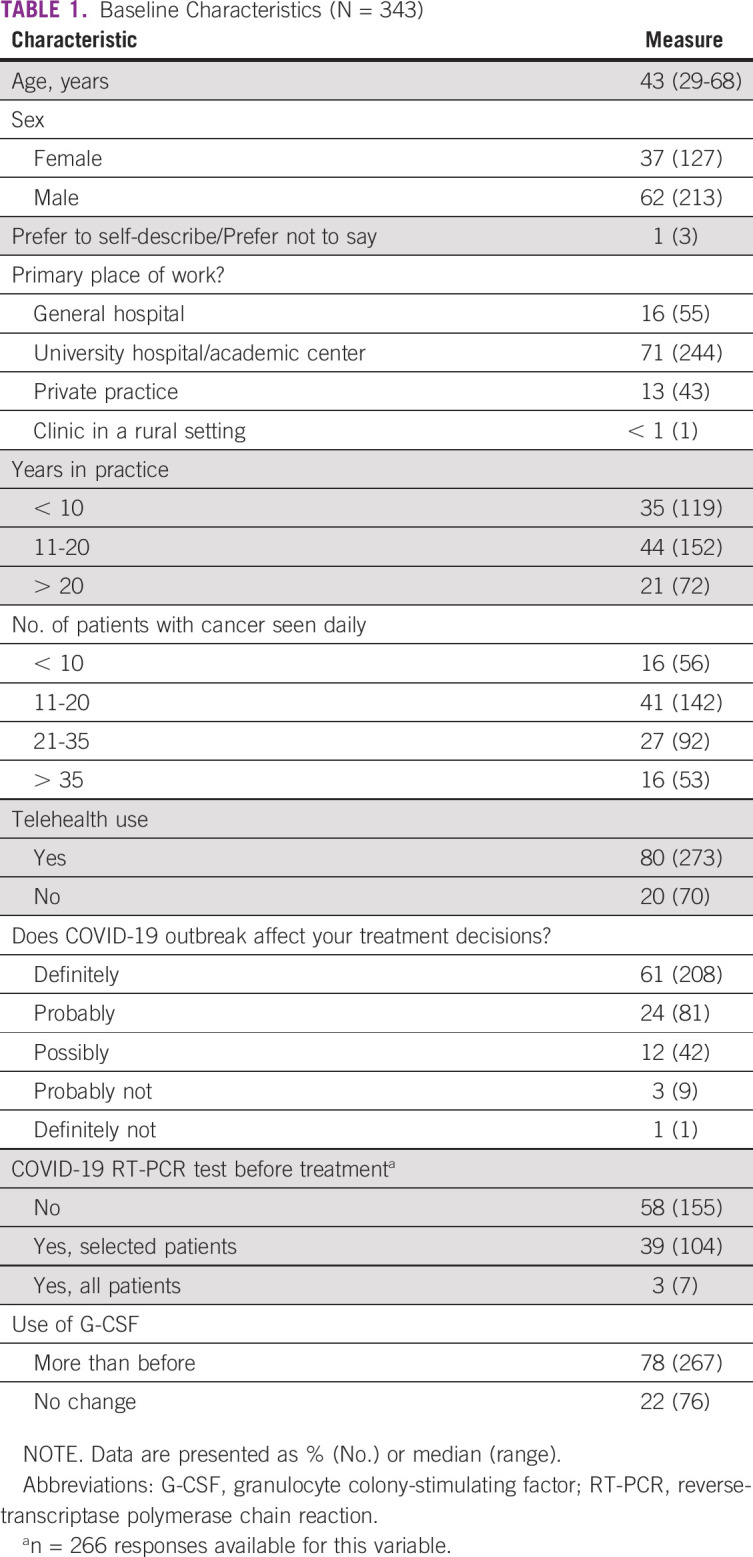
Baseline Characteristics (N = 343)

**TABLE 2:**
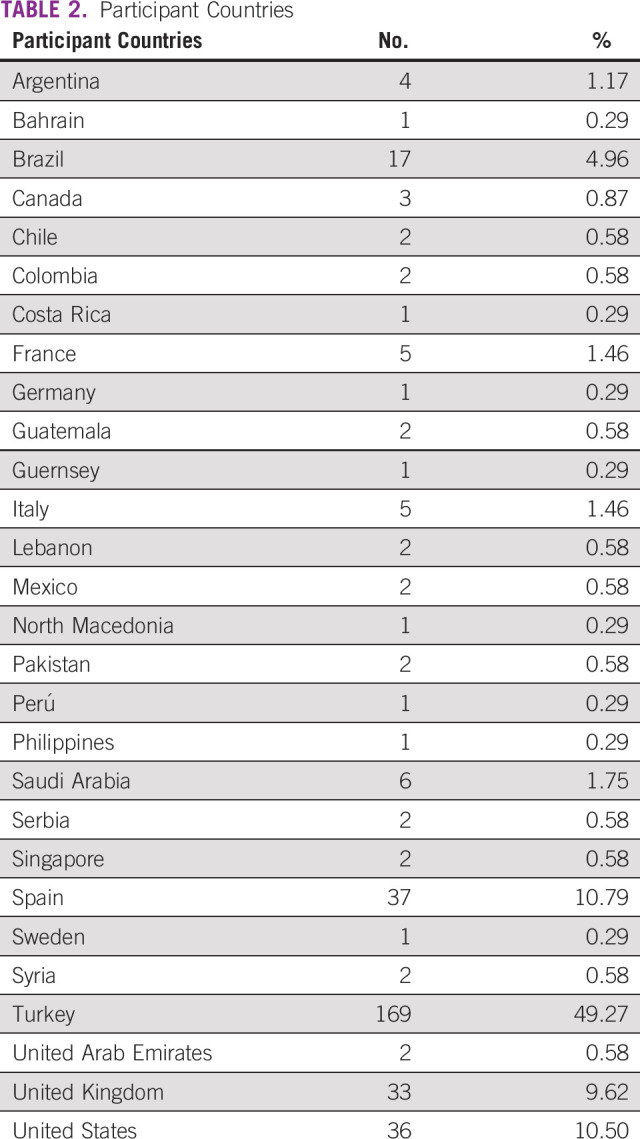
Participant Countries

### Readiness Measures

**FIG 1:**
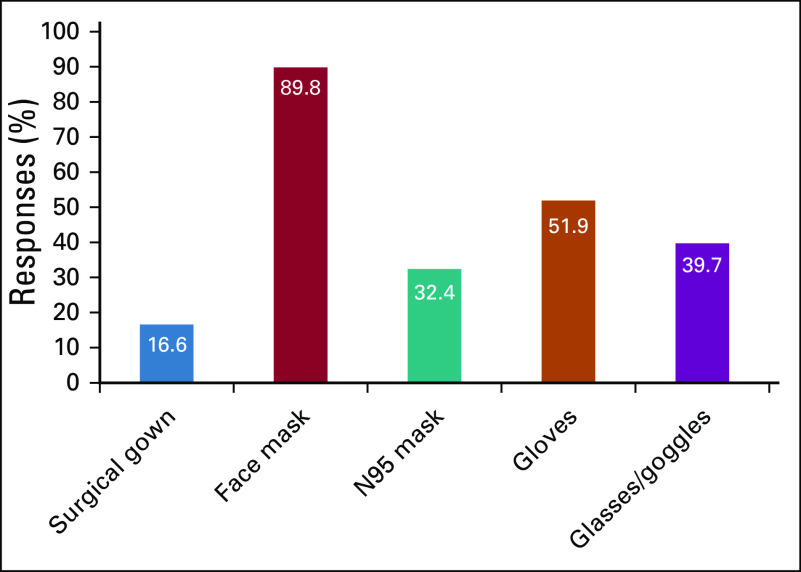
Respondent answers to “What precautions are you taking for yourself during clinical practice?”

Overall, 43% of participants cared for ≥ 20 patients daily during the pandemic, and 16% saw ≥ 35 patients. The use of telemedicine among the participants was quite common (80%). All participants stated that they were consistently using personal protective equipment (PPE), of which surgical mask (90%), gloves (52%), and glasses (39%) were most frequently used. N95 mask usage rate was found to be 33% (Fig 1). Because the question “Do you perform COVID-19 RT-PCR test before the treatment” was added to the questionnaire after the initial inception of the questionnaire, only 266 answers were received. Although 58% stated that they did not perform routine testing, 39% stated that they performed reverse-transcriptase polymerase chain reaction (RT-PCR) tests in selected patients and 3% in all patients.

### Participant Attitudes

**FIG 2:**
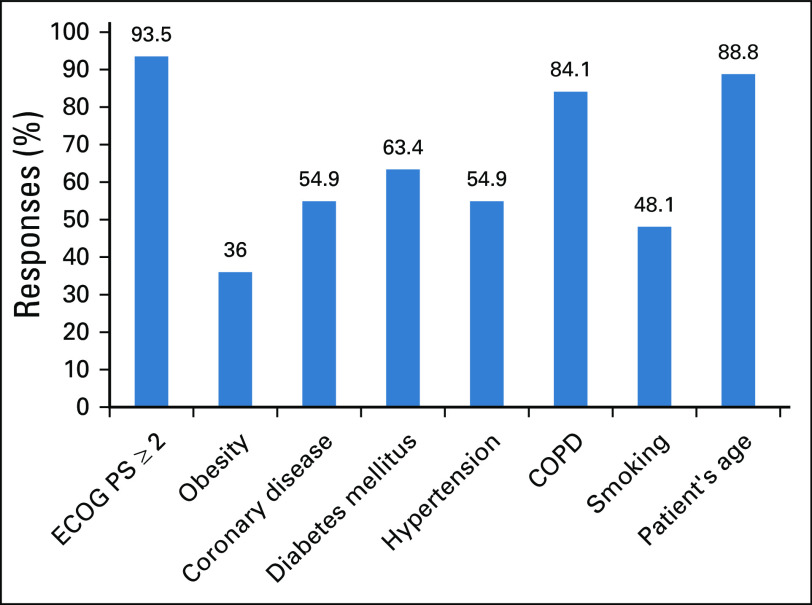
Respondent answers to “Which comorbidities do you think affect your treatment decisions?” COPD, chronic obstructive pulmonary disease; ECOG PS, Eastern Cooperative Oncology Group performance status.

**FIG 3:**
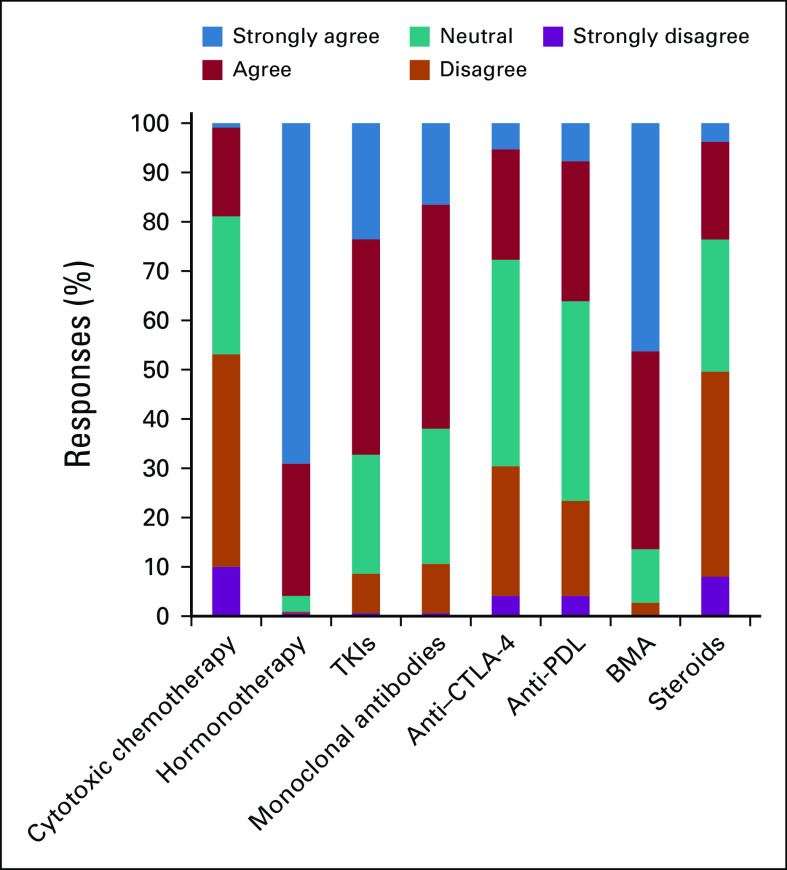
Respondent answers to “During the COVID-19 outbreak, do you think the following treatments are safe?” BMA, bone-modifying agents; TKI, tyrosine kinase inhibitor.

**FIG 4:**
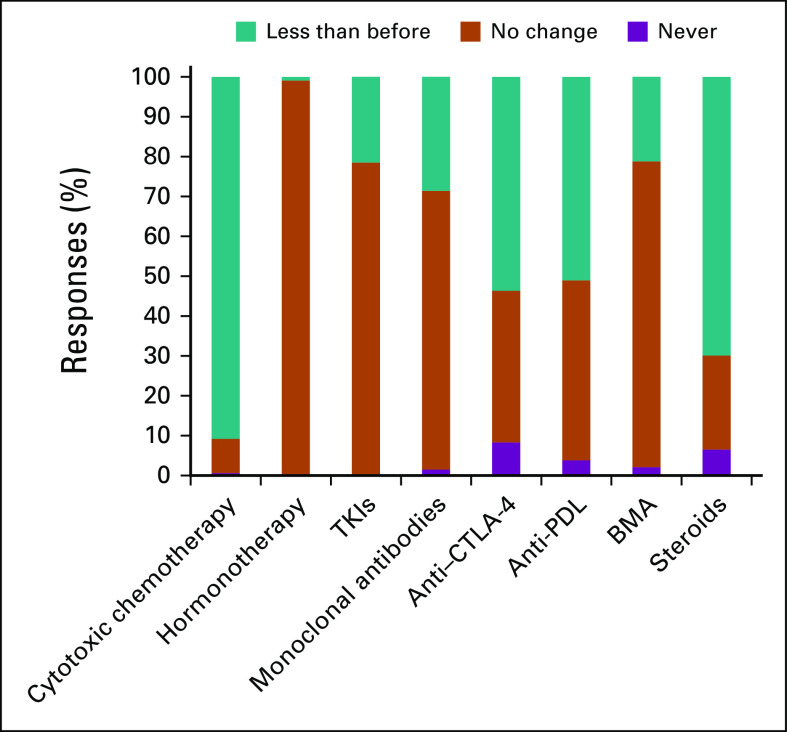
Respondent answers to “Comparing with your previous practice would you recommend the following treatments during the COVID-19 outbreak?” BMA, bone-modifying agents; TKI, tyrosine kinase inhibitor.

When asked about factors affecting treatment decision making, participants stated patient age and concomitant diseases were influential factors (81% and 92%, respectively; Fig 2). Regarding perceptions about the safety of antineoplastic therapy, hormonal treatments and tyrosine kinase inhibitors (TKIs) were considered to be relatively safe, but cytotoxic chemotherapy and immune therapies were considered less safe or unsafe (Fig 3). Most participants stated that during the pandemic they would use less chemotherapy, anti–CTLA-4 antibody, anti–PD-1 or PD-L1 antibodies, and corticosteroids. However, participants did not express alterations in prescribing patterns for hormonal therapies, TKIs, and bone-modifying agents (Fig 4). A total of 78% of the participants stated that they would use granulocyte colony-stimulating factor (G-CSF) more frequently.

**FIG 5:**
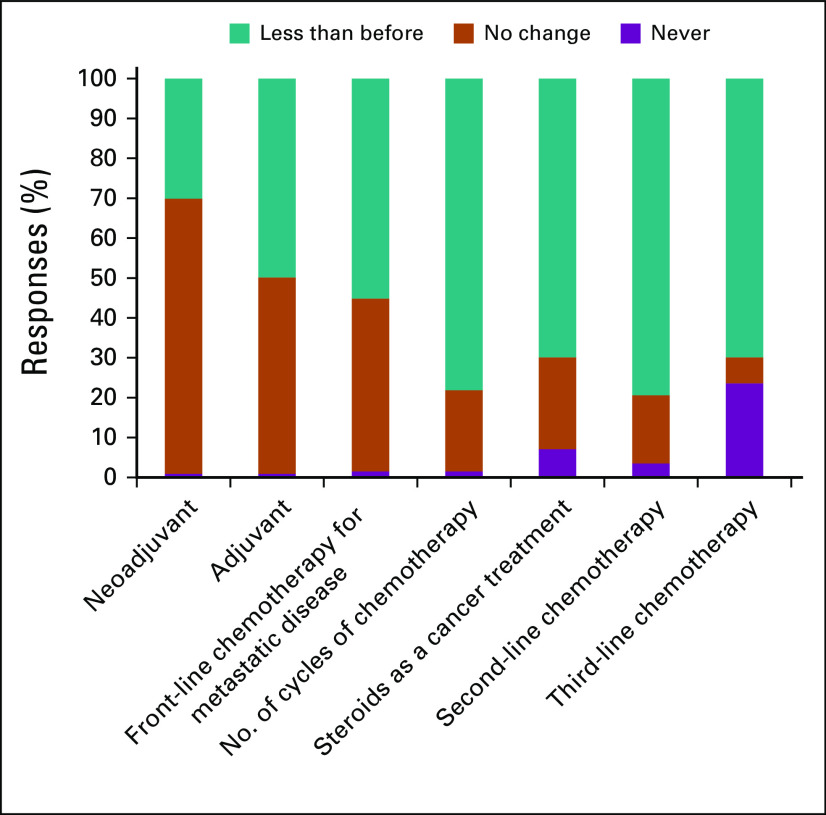
Respondent answers to “Comparing with your previous practice would you change your treatment algorithms for the following settings during the COVID-19 outbreak?”

In general, the decision to reduce use across all therapy categories was expressed by participants. The degree of therapy reductions was less pronounced for use of therapy in the neoadjuvant and adjuvant setting. Second- and third-line treatment use for metastatic disease was dramatically reduced across survey participants (Fig 5).

**FIG 6:**
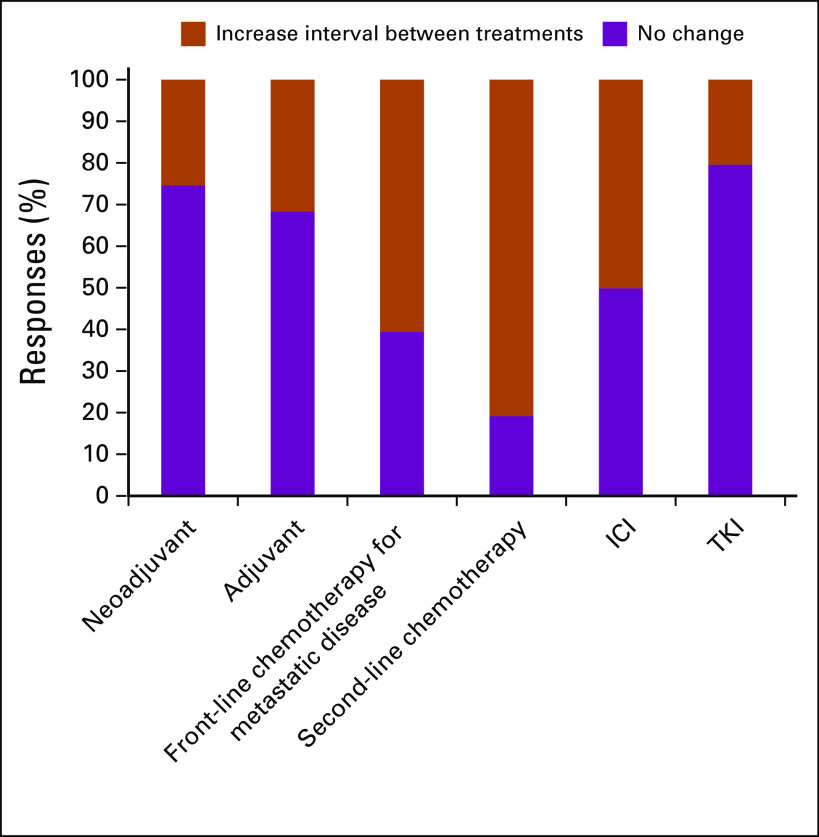
Respondent answers to “Comparing with your previous practice would you change dose density for the following settings during the COVID-19 outbreak?” ICI, immune checkpoint inhibitor; TKI, tyrosine kinase inhibitor.

**FIG 7:**
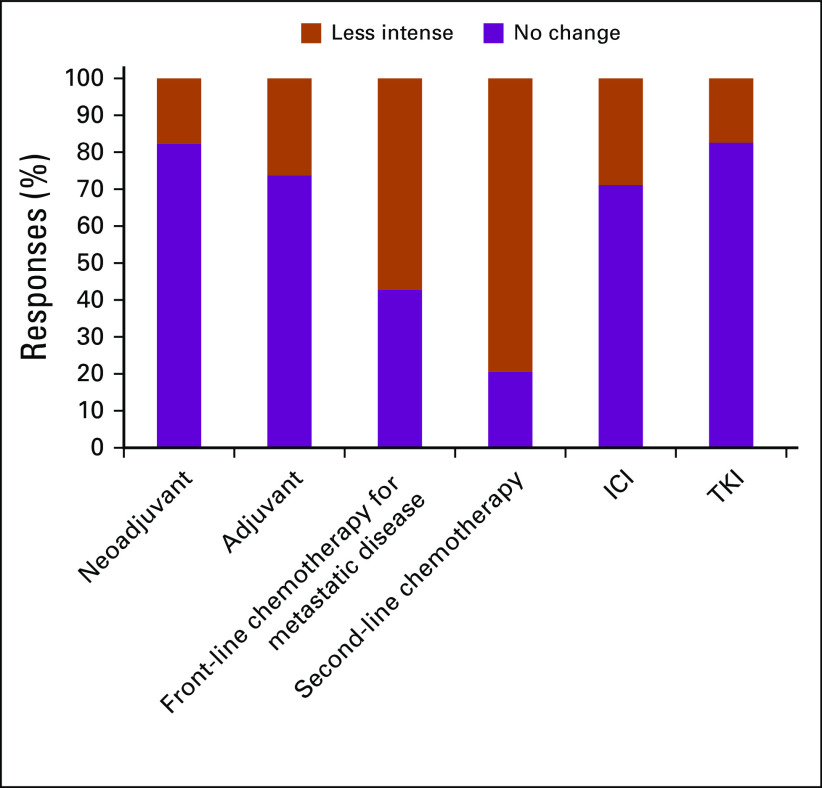
Respondent answers to “Comparing with your previous practice would you change dose intensity for the following settings during the COVID-19 outbreak?” ICI, immune checkpoint inhibitor; TKI, tyrosine kinase inhibitor.

We asked participants whether they would modify systemic treatment dosing, schedules, and context of use (Figs 6 and 7). No significant differences in demographic, preventive, or practice-related data were seen among participants of different nationalities.

## DISCUSSION

The COVID-19 pandemic has resulted in changes in the delivery of cancer care.^16,17,24,26,27,29-31^ Since its emergence, COVID-19 has rapidly crossed all borders and affected health care networks globally. Health care systems and medical professionals have been propelled to respond to the evolving and complex situation, with some of them being rapidly overwhelmed by a sudden high number of cases requiring health care resource reallocation. Given early data suggesting that patients with cancer may be at substantially higher risk of COVID-19–related complications, medical oncologists face unique challenges in continuing to meet the needs of both patients and staff during this unprecedented pandemic. This survey provides important context of the readiness measures and perceptions of medical oncologists during the initial stages of the pandemic.

Our results demonstrate that, although oncologists are trying to continue treating their patients on the basis of guidelines, despite the lack of evidence regarding COVID-19–related risk at the time of this study, they have made important modifications in usual practice. Although neoadjuvant and adjuvant treatments in curative settings are less affected, a decrease in treatment of metastatic disease is expected based on our survey. Hormonal treatments are generally considered safer, and there is no anticipated change in delivery of these therapies. On the other hand, there is no consensus about the safety of monoclonal antibodies and immunotherapies among oncologists surveyed. Although most recent data suggest that giving immune checkpoint inhibitors (ICIs) to COVID-19–positive patients with cancer is safe,^8,18,32^ it is highly probable that the oncological community has acted with caution and reserve regarding initiation or maintenance of such treatments during the last months, potentially affecting patient outcomes.

Telemedicine has been implemented at a low rate over the decade, but it has become increasingly useful while mobility is reduced and social distancing is mandated for pandemic control.^33,34^ In our survey, 80% of the participants stated that they used telemedicine in some form during this pandemic. However, the adaptation of the legal infrastructure and reimbursement systems for telemedicine are still ongoing. In addition, it is necessary to keep in mind some difficulties. Limited use of phones, smartphones, or internet access may be a barrier, especially in rural areas, as well as for elderly patients. We will need to develop strategies to overcome these issues with care delivery.

SARS-CoV-2 is a highly transmissible virus, and health care professionals have been at the forefront of workers with the highest risk of infection. Recently, the Infectious Diseases Society of America published a guideline on PPE that should be used for the protection of health care workers.^35^ The vast majority of survey participants stated that they used surgical masks. Although only 32% of respondents described using N95 masks, the survey did not capture the proportion of respondents who had access to N95 masks while caring for patients known to have COVID-19, which is recommended, especially while doing invasive procedures such as intubation, bronchoscopy, and any airway-related manipulations.^36^ Unfortunately, severe shortages of PPE globally have created significant challenges.^35,37^

A significant proportion of patients with COVID-19 are asymptomatic, increasing the risk of recommending active cancer treatment during the pandemic.^38-40^ Furthermore, the PCR-based test used for the diagnosis of COVID-19 is currently of suboptimal accuracy; in some cases where radiologic COVID-19 is considered, the PCR test may be negative.^41^ However, there is no recommendation as to whether routine PCR testing should be required. Testing availability and defining populations in which screening tests should be performed for asymptomatic patients and diagnostic tests for symptomatic patients remains a challenge globally. Also, developing workflows to operationalize testing in a safe manner for patients and health care works will be critical in mitigating viral spread.

The case fatality rate increases in the elderly population and in patients with comorbidities such as diabetes mellitus, hypertension, and cancer.^2,10,13,20,27,42-44^ However, current data regarding cancer and COVID-19 remain elusive. In a recent meta-analysis by Desai et al,^45^ the overall pooled prevalence of cancer in patients with COVID-19 was 2.0%, suggesting at least a doubling of the risk compared with the general population. Given the heterogeneity present among oncology patients, population-based estimates may not estimate an individual’s risk. When making treatment decisions in patients with cancer, oncologists consider a patient's age, performance status, and concomitant diseases, among many factors. This individualized approach will be central to carefully evaluating the risk/benefit profile of anticancer treatments during the pandemic. In our study, 80% of participants stated that age would affect their treatment decisions, and 90% stated that the presence of concomitant diseases would do so. Considered together, age, Eastern Cooperative Oncology Group (ECOG) performance status ≥ 2, or the presence of chronic obstructive pulmonary disease (COPD) influenced > 80% of the participants’ treatment decisions.

Conventionally, curative cancer treatment often involves neoadjuvant and/or adjuvant systemic treatment. Although there is a modest decrease in the use of neoadjuvant therapy compared with the prepandemic period—which may correspond to a delay from some surgical interventions during the pandemic—and a marked 50% reduction in adjuvant treatment use, treatment practices are being carried out with relative preservation of dose density and intensity in the curative setting. For patients with metastatic disease, 60% of the participants stated that they would offer first-line treatment less frequently, and in case of systemic treatments, 80% of the participants stated that they would decrease the number of cycles of chemotherapy to be given.

During the pandemic, it is perceived as essential to administer curative treatments as much as possible. However, in the case of treatment regimens for which the incremental benefits are low and the risk of infection is high, such as second- and third-line therapy for metastatic disease, it sounds more reasonable to colleagues to curtail use to maximize survival in a patient population that may be more debilitated at baseline. Largely, these decisions are highly individualizing. It has become a priority to discuss and refine the multiplicity of parameters for decision making within our community as well as with the patients. The magnitude of expected clinical benefit should be evaluated for each intervention. Although some groups have attempted to develop standardized guidelines, these are not evidence based, given the unprecedented nature of the pandemic. Such evidence-based statements will need multivariable analyses of extremely large numbers of patients with cancer.

Interestingly, hormonal treatments are generally considered safe. Because there are sex differences in susceptibility and vulnerability to COVID-19, several hypotheses related to the androgen pathway have been proposed. Two different studies suggest that the use of antiandrogens may be protective for COVID-19 in patients with prostate cancer.^46,47^ In this survey, a significant part of the participants (96%) stated that they considered hormonal treatments safe.

Immunotherapy has rapidly become part of the standard treatment protocols for many cancers, including melanoma, lung, kidney, and bladder. However, ICIs can cause severe immune-mediated toxicity, such as pneumonitis, colitis, hepatitis, and endocrine disorders.^48^ Because management of ICI toxicity rarely requires the use of immunosuppressive steroids, we see some reluctance among clinicians to prescribe ICIs during the COVID-19 pandemic. There is concern that ICIs can increase the severity of the disease because of their immunomodulatory properties.^42^ Although almost half of the participants were reticent about whether they were safe or not, one-third of them stated that they did not think it was safe. However, there is currently paucity of data regarding ICIs and COVID-19. The 2020 AACR national meeting featured COVID-19 and cancer special sessions. Data presented by Barlesi et al^19^ from 137 patients with cancer and COVID-19 who were treated at Gustave Roussy showed that an ECOG performance status > 1, hematologic malignancies, and chemotherapy within the past 3 months were associated with worse outcomes; however, immunotherapy or targeted agents in the past 3 months did not associate with the deterioration of the COVID-19 clinical course.^19^ In contrast to this study, Robilotti et al^49^ recently presented results of 423 patients with cancer and COVID-19 disease from Memorial Sloan Kettering Cancer Center (MSKCC). In the study, being > 65 years of age and undergoing treatment with ICIs within 90 days were predictors for hospitalization and severe disease. However, in a recent study, which was also from MSKCC, including 69 patients with COVID-19 and lung cancer, PD-1 blockade was not associated with the severity of COVID-19.^32^ The US Food and Drug Administration has approved doses of nivolumab administered every 4 weeks and pembrolizumab every 6 weeks. This will be more convenient in terms of reducing the frequency of patients coming to the hospital. In the study presented by Zhang et al,^14^ having cancer treatment in the last 14 days was found to be associated with a more serious clinical course of COVID-19. In the follow-up of 124 patients who received ICIs, only 1 patient had COVID-19, and their clinical course was mild. In another recently published large cohort study from China, hematologic malignancy, lung cancer, or metastatic cancer (stage IV) were associated with increased frequency of severe events. There were no differences regarding the severity of COVID-19 between patients with nonmetastatic cancer and patients without cancer.^17^ The first results of the TERAVOLT (Thoracic Cancers International COVID-19 Collaboration) were also presented during AACR 2020. Data from 200 patients with thoracic cancer were examined, revealing that the presence of COPD was associated with hospitalization and multiple comorbidities associated with hospitalization and death risk. However, tumor type and cancer therapy did not affect survival.^15^

In our survey, 80% of oncologists stated that they used more G-CSF than before. Using G-CSF can protect patients from hospitalization through reducing the risk of neutropenic fever. Patients who required intensive care unit admission because of COVID-19 showed a higher percentage of GM-CSF+ CD4+ T cells, suggesting excessive activation of the immune response by G-CSF may promote the development of lung injury.^50^ Therefore, although G-CSF may reduce hospitalization from neutropenic complications, it carries a theoretical risk of promoting pulmonary injury and aggravating the COVID-19 course.^22,50^ Given the absence of clinical data to resolve this, evidence is needed to clarify how GM-CSF modulates the global risk of patients.

Our study has several limitations. The limited number and format of questions does not provide an in-depth quantitative analysis of some common clinical practices. However, we expected the brevity would increase response rate and reliability. In addition, we cannot confirm that all participants were medical oncologists, and although conducted globally, the survey does not evenly represent all countries This report serves as a pilot study to learn general approaches and immediate reactions of oncologists at this point in the COVID-19 pandemic and to identify difficulties and uncertainties in clinical decision making that would benefit from clearer guidance on the basis of reliable data.

Many uncertainties exist with regard to COVID-19 and infection in patients with cancer. The risk/benefit ratio of the decisions we make and the expected benefit of everything we do have become essential arguments and limiting factors at the time of COVID-19. Counterintuitively, the absolute benefit of an adjuvant therapy can sometimes be modest in a curative setting, while it can be major in some metastatic cancers where the palliative versus curative impact of immunotherapy can be disputed. Ongoing research is essential to improve our understanding of the disease and optimize health care delivery strategies for patients with cancer. This survey provides an important context to assess current physician readiness and attitudes about care delivery during the pandemic. The COVID-19 pandemic has affected, and continues to affect, both patients and oncologists in a variety of ways. As in all oncological practice, it is critical that each patient be evaluated on an individual basis, and the risk/benefit ratio of any proposed therapy must be evaluated by a patient’s treating oncologist.^28^ Although ESMO and ASCO have published general guidelines to oncological practice, it is impossible to provide recommendations for each clinical scenario.^51^ For this reason, it is more important than ever that colleagues continue to systematically discuss their patients in tumor board settings. In addition, it is essential that the oncology community gather comprehensive, rigorous data to further improve the clinical decision-making process during this unprecedented moment.^52^ The COVID-19 and Cancer Consortium, a multicenter, voluntary registry collecting and examining data on risk factors and outcomes of patients with cancer who develop COVID-19, will play an important role in understanding how baseline characteristics and systemic treatment modalities affect the risk of severe COVID-19.^29,30^ We hope, as our experience, collaboration, and knowledge sharing improve, that we will be able to more effectively manage this outbreak with more evidence-based interventions and treatments.
